# Towards defining morphologic parameters of normal parous and nulliparous breast tissues by artificial intelligence

**DOI:** 10.1186/s13058-022-01541-z

**Published:** 2022-07-11

**Authors:** Joshua Ogony, Thomas de Bel, Derek C. Radisky, Jennifer Kachergus, E. Aubrey Thompson, Amy C. Degnim, Kathryn J. Ruddy, Tracy Hilton, Melody Stallings-Mann, Celine Vachon, Tanya L. Hoskin, Michael G. Heckman, Robert A. Vierkant, Launia J. White, Raymond M. Moore, Jodi Carter, Matthew Jensen, Laura Pacheco-Spann, Jill E. Henry, Anna Maria Storniolo, Stacey J. Winham, Jeroen van der Laak, Mark E. Sherman

**Affiliations:** 1 Quantitative Health Sciences, Mayo Clinic College of Medicine, Rochester and Jacksonville, MN and FL USA; 2 Department of Pathology, Radboud University Medical Center, Nijmegen, The Netherlands; 3 Radboud Institute for Health Sciences, Radboud University Medical Center, Nijmegen, The Netherlands; 4 Department of Cancer Biology, Mayo Clinic College of Medicine, Jacksonville, FL USA; 5 Department of Surgery, Mayo Clinic College of Medicine, Rochester, MN USA; 6 Department of Oncology, Mayo Clinic, Rochester, MN USA; 7 Division of Epidemiology, Mayo Clinic College of Medicine, Rochester, MN USA; 8 Department of Laboratory Medicine and Pathology, Mayo Clinic College of Medicine, Rochester, MN USA; 9 Susan G. Komen Tissue Bank at the IU Simon Cancer Center, Indiana University School of Medicine, Indianapolis, USA; 10 Center for Medical Image Science and Visualization, Linköping University, Linköping, Sweden; 11 Division of Epidemiology, Department of Health Sciences Research, Mayo Clinic, 4500 San Pablo Rd, Jacksonville, FL 32224 USA

**Keywords:** Breast cancer, Involution, Postpartum, Risk, Epidemiology, Terminal duct lobular units

## Abstract

**Background:**

Breast terminal duct lobular units (TDLUs), the source of most breast cancer (BC) precursors, are shaped by age-related involution, a gradual process, and postpartum involution (PPI), a dramatic inflammatory process that restores baseline microanatomy after weaning. Dysregulated PPI is implicated in the pathogenesis of postpartum BCs. We propose that assessment of TDLUs in the postpartum period may have value in risk estimation, but characteristics of these tissues in relation to epidemiological factors are incompletely described.

**Methods:**

Using validated Artificial Intelligence and morphometric methods, we analyzed digitized images of tissue sections of normal breast tissues stained with hematoxylin and eosin from donors ≤ 45 years from the Komen Tissue Bank (180 parous and 545 nulliparous). Metrics assessed by AI, included: TDLU count; adipose tissue fraction; mean acini count/TDLU; mean dilated acini; mean average acini area; mean “capillary” area; mean epithelial area; mean ratio of epithelial area versus intralobular stroma; mean mononuclear cell count (surrogate of immune cells); mean fat area proximate to TDLUs and TDLU area. We compared epidemiologic characteristics collected via questionnaire by parity status and race, using a Wilcoxon rank sum test or Fisher’s exact test. Histologic features were compared between nulliparous and parous women (overall and by time between last birth and donation [recent birth: ≤ 5 years versus remote birth: > 5 years]) using multivariable regression models.

**Results:**

Normal breast tissues of parous women contained significantly higher TDLU counts and acini counts, more frequent dilated acini, higher mononuclear cell counts in TDLUs and smaller acini area per TDLU than nulliparas (all multivariable analyses *p* < 0.001). Differences in TDLU counts and average acini size persisted for > 5 years postpartum, whereas increases in immune cells were most marked ≤ 5 years of a birth. Relationships were suggestively modified by several other factors, including demographic and reproductive characteristics, ethanol consumption and breastfeeding duration.

**Conclusions:**

Our study identified sustained expansion of TDLU numbers and reduced average acini area among parous versus nulliparous women and notable increases in immune responses within five years following childbirth. Further, we show that quantitative characteristics of normal breast samples vary with demographic features and BC risk factors.

**Supplementary Information:**

The online version contains supplementary material available at 10.1186/s13058-022-01541-z.

## Introduction

Breast terminal duct lobular units (TDLUs) are structures where breastmilk is produced and where most human breast cancer (BC) precursors arise (i.e., benign breast disease (BBD)) [1, 2]. TDLUs are comprised of acini and ducts lined by luminal epithelium and myoepithelium, surrounded by stroma containing vessels and immune cells. Over the life course, TDLUs undergo dramatic alterations related to pregnancy, lactation, and postpartum involution (PPI) [3–7], a dramatic process that restores baseline morphology after weaning, and age-related involution, a gradual process that may begin as early as the fourth decade [8, 9]. PPI is associated with massive cell death and inflammation, which is superimposed on the proliferative changes of pregnancy, and is followed by wound healing and re-modeling to restore baseline breast architecture [3–7]. Preclinical, epidemiological, and clinical studies implicate dysregulated PPI in the pathogenesis of clinically aggressive breast cancers developing in the postpartum period [3–7, 10–13]; consequently, defining morphologic characteristics of normal postpartum breast tissue and determining the associations of BC risk factors with these features may provide a useful reference for comparison with BBD biopsies performed for clinical diagnosis among young women. Previously, we have reported that higher levels of age-related involution of background TDLUs included in BBD biopsies is related to lower BC risk [14, 15]. We propose that by defining physiological changes in TDLUs during the postpartum period and comparing these features with nulliparous women, we may be able to define features related to BC risk in future studies.

Most studies comparing the morphology of parous and nulliparous breast tissues have evaluated normal appearing TDLUs within clinical biopsies performed for indications (e.g., abnormal mammograms or BC), included limited epidemiological data and used visual assessment and/or basic morphometry to characterize tissues [6, 8, 14, 15]. To address this potential limitation, we applied two strategies: 1) we analyzed normal breast tissues donated for research (i.e., not removed to diagnose pathology) to the Komen Tissue Bank (KTB) [16], which collects extensive BC risk factor data from participants and 2) we analyzed tissue histology using three independent methods: a validated automated pathology Artificial Intelligence (AI) method [17], visual assessment, and morphometry [8, 15], thus providing a rigorous approach. Although diagnostic pathology relies mainly on visual qualitative assessments, rapid implementation of digital pathology into clinical practice offers opportunities to apply computational pathology methods to enhance diagnosis [18]. Accordingly, developing quantitative reference ranges for morphologic features, analogous to blood-based clinical tests, may have future importance [19].

## Methods

### Study population

KTB was established in 2007 to acquire questionnaires, blood, and normal breast tissues from consented volunteers [16]. Donors provide multiple breast tissue cores that are snap-frozen or fixed and paraffin-embedded. The KTB uses standardized tissue sampling and our previous analysis of samples (*n* = 50) demonstrated that adjusting for tissue area in images of hematoxylin and eosin (H&E)-stained sections does not impact conclusions (unpublished). The KTB protocol was approved by the Institutional Review Board at Indiana University. The current project was also approved by the Mayo Clinic Institutional Review Board.

To compare histologic features of breast tissues of younger parous and nulliparous women, we obtained data for all 1,082 KTB donors enrolled from 2009 to 2018 and aged ≤ 45 years at enrollment, including 801 nulliparas and 281 parous women who had given birth within 10 years of tissue acquisition. We excluded women who did not self-identify as white or African American (29 parous, 96 nulliparous), women who were found to be above age 45 years at donation on further review (17 parous, 52 nulliparous), women whose samples did not contain evaluable TDLUs (36 parous, 63 nulliparous), women with a history of cancer (9 parous, 11 nulliparous), and women who had undergone an oophorectomy (7 parous, 5 nulliparous). For 32 repeat donors (3 parous and 29 nulliparous), only the first tissue donation was analyzed. The final sample set analyzed by AI and visual morphometry included 725 women, of whom 180 were parous (149 white, 31 African American; mean age = 35 years) and 545 were nulliparous (479 Caucasian, 66 African American; mean age = 25 years.

### Exposure assessment

Women completed self-administered BC risk factor questionnaires assessing age at donation, race, ethnicity, age at first period, menopausal status, age at first birth, number of live births, any relatives with a history of breast/ovarian cancer, medical conditions, weight and height (assessed as body mass index (BMI) (Kg/m^2^)), smoking and ethanol consumption. A supplementary early life questionnaire assessing onset of puberty, dates of live births, and breastfeeding history (total months across all pregnancies) was collected https://komentissuebank.iu.edu/donate-tissue/docs/early-life-questionnaire-letter-a071.pdf.

### Tissue analysis by AI and by visual review with morphometry

We applied previously validated automated AI methods to quantitate features related to scanned whole slide images (Aperio ScanScope XT Slide Scanner, Leica Biosystems) at 20X with resolution of 0.495 × 0.495  μm^2^ per pixel or specific to TDLUs (Additional file 1: Fig. 1). Whole slide metrics assessed, included: TDLU count and adipose tissue fraction; TDLU-specific features, included: mean acini count/TDLU; mean dilated acini; mean average acini area; mean “capillary” area; mean epithelial area; mean ratio of epithelial area versus intralobular stroma; mean mononuclear cell count; mean fat area proximate to TDLUs and TDLU area. Mononuclear cell counts refer to all cells with small round nuclei, which may represent lymphocytes, plasma cells, macrophages, and less common immune cell types (e.g., mast cells). Previously, we found that our AI measurements agreed well with results of a four-member panel using a 6-tier classification of TDLU involution (unweighted kappa = 0.747 ± 0.01) (Additional file 1: Fig. 1) [17].

A trained reviewer (JO) independently assessed morphometric features of TDLUs using a validated method [8, 15, 17]. We limited morphometric analysis to normal appearing TDLUs and excluded TDLUs showing subtle features of benign breast disease, represented mainly by dilatation of acini two- to three-fold or focal metaplastic or proliferative changes. Total normal TDLUs per section were counted and ≤ 10 TDLUs first encountered per section in scanning were evaluated for acini number/TDLU scored categorically (1 = 1–9, 2 = 10–19, 3 = 20–29, 4 = 30–39, 5 = 40–49, 6 =  > 50), and TDLU span in um [8, 15, 17]. Data were analyzed per participant as the mean acini counts/TDLU and median TDLU span. A pathologist (MES) masked to other data, assessed images that contained ≥ 5 TDLUs (147 parous and 381 nulliparous donors) for “TDLU inflammation”, subjectively assessed as hypercellularity versus surrounding TDLUs, plasma cells (present if ≥ 3 per section), and dilated acini. AI and morphometric features were highly correlated: TDLU counts (r = 0.84; *p* < 0.0001); acini counts (*p* = 0.80; *p* < 0.0001). AI mononuclear cell counts were associated with visual assessment of inflammation (mean/maximum counts per TDLU for inflamed:118.2/444.0 versus not inflamed: 82.0/233.5, respectively; both comparisons *p* < 0.001). Our AI method was not trained to identify plasma cells.

### Statistical analysis

We compared epidemiologic characteristics between parous and nulliparous women, and between white and African American women, using a Wilcoxon rank sum test (continuous and ordinal variables) or Fisher’s exact test (categorical variables). Histologic features (assessed by AI or visually) were compared between nulliparous and parous women (overall and by time between last birth and donation [ recent birth: ≤ 5 years versus remote birth: > 5 years]) using multivariable regression models (linear regression, negative binomial regression, proportional odds logistic regression, and binary logistic regression) appropriate to the nature of the specific histologic feature (continuous, count, ordinal or dichotomous, respectively; see table footnotes). Dichotomization at 5 years was chosen to reflect peak postpartum BC risk [13]. We performed exploratory analysis to assess variability among women who donated ≤ 5 years of last birth. For linear regression models, data transformations were applied to account for distributional skewness as needed (see table footnotes), and additive effects on the mean value of the histologic feature (i.e., parous minus nulliparous) were estimated with 95% confidence intervals (CIs). For negative binomial regression models, multiplicative effects on mean values of histologic features were estimated with 95% CIs. For proportional odds logistic regression models, odds ratios (ORs) and 95% CIs were estimated and are interpreted as the multiplicative increase in the odds of a higher category for parous versus nulliparous women. For logistic regression models, ORs and 95% CIs were estimated and are interpreted as the multiplicative increase in the odds of histologic features among parous versus nulliparous women. Multivariable models were initially adjusted for age at donation, and then further adjusted for any characteristic that differed between groups (*p* value < 0.15). Comparisons of AI features between white and African American women were made following this same multivariable regression analysis strategy. A Bonferroni correction for multiple testing was applied separately to AI analyses (11 tests, *p* values < 0.0045 considered as statistically significant) and for visual/morphometric assessments (6 tests, *p* values < 0.0083 considered as statistically significant).

We also performed exploratory analyses of determinants of TDLU number, dilated acini and mononuclear cells count (AI) or inflammation (morphometry) separately among parous and nulliparous women using regression models and compared AI histologic features by race. For reference, analyses among parous women after Bonferroni correction would require *p* ≤ 0.0036 and among nulliparas, *p* ≤ 0.0056 to achieve statistical significance. All statistical tests were two-sided and were performed using SAS (version 9.4; SAS Institute, Inc., Cary, North Carolina).

## Results

### Characteristics of KTB breast tissue donors

Participants included 545 nulliparous and 180 parous women, of whom 103 had their most recent birth within 5 years and 77 more than 5 years previously (median time since birth of 4 years). Characteristics are presented in Table 1. Compared with nulliparas, parous women were older (median 35 years versus 25 years; p < 0.001), more frequently reported any family history of breast/ovarian cancer (61.9% vs 49.3%, *p* = 0.006) and were marginally heavier (median BMI of 27.0 versus 25.4; *p* = 0.062). Compared with white women, African American donors were older (median age of 33 versus 27 years (*p* < 0.001)), heavier (median BMI = 31.6 versus 25.1 kg/m^2^; *p* < 0.001), reported less frequent ethanol use (59.8% versus 73.9%; *p* = 0.005); earlier age at menarche (*p* < 0.001), and, among parous women, longer interval from birth to donation (median 6.0 versus 3.7 years; *p* = 0.012) (Additional file 1: Table 1).

**Table 1:** Comparisons of characteristics between parous and nulliparous women

Variable	*N*	Parous women (*N* = 180)	*N*	Nulliparous women (N = 545)	*p* value
Age of donation (years)	180	35 (22, 45)	545	25 (18, 45)	< 0.001
Ethnicity (Hispanic/Latino)	180	13 (7.2%)	544	39 (7.2%)	1.00
Race	180		545		0.10
Caucasian		149 (82.8%)		479 (87.9%)	
African American		31 (17.2%)		66 (12.1%)	
BMI	180	27.0 (14.3, 62.9)	545	25.4 (13.3, 63.2)	0.062
Current smoking	166	5 (3.0%)	530	31 (5.8%)	0.17
Current drinking	179	125 (69.8%)	542	394 (72.7%)	0.50
Age at first period (years)	180	12.5 (8, 19)	545	12 (8, 18)	0.44
Menstrual status	180		544		0.82
Pre-menopausal		173 (96.1%)		525 (96.5%)	
Post-menopausal		4 (2.2%)		11 (2.0%)	
Uterine ablation		3 (1.7%)		8 (1.5%)	
Number of live births	180		N/A	N/A	N/A
1		61 (33.9%)			
2		83 (46.1%)			
3		31 (17.2%)			
4		4 (2.2%)			
5		1 (0.6%)			
Time since last birth (years)	180	4.0 (0.1, 11.0)	N/A	N/A	N/A
Age at first birth (years)	180	28 (16, 41)	N/A	N/A	N/A
History of breastfeeding	180	156 (86.7%)	N/A	N/A	N/A
Total months breastfeeding	156	9 (1, 90)	N/A	N/A	N/A
Total months breastfeeding (with no breastfeeding considered to be 0 months)	180	6 (0, 90)	N/A	N/A	N/A
Relative with breast/ovarian cancer	168	104 (61.9%)	511	252 (49.3%)	0.006

The sample median (minimum, maximum) is given for continuous variables. *p* values result from a Wilcoxon rank sum test (continuous variables) or Fisher’s exact test (categorical variables)

### Comparison of breast tissues of parous versus nulliparous women

AI analyses revealed that tissues of parous women contained significantly higher TDLU counts and acini counts, more frequent dilated acini, higher mononuclear cell counts in TDLUs and smaller acini area per TDLU than nulliparas (all multivariable analyses *p* < 0.001; significant after multiple comparison adjustment) (Table 2). Using visual morphometric assessment, parous women had significantly higher TDLU counts (*p* < 0.001), and suggestively, but non-significantly increased plasma cells (*p* = 0.20) and inflammation (*p* = 0.14) (Additional file 1: Table 2). Associations remained after adjusting for age and other potential confounding variables.

**Table 2:** AI data for parous versus nulliparous women

Outcome	Parous women (*N* = 180)		Nulliparous women (*N* = 545)		Association measure	Comparison between parous women and nulliparous women (reference group)			
						Age-adjusted analysis		Adjusting for age, race, BMI, percent fat, and relative with breast or ovarian cancer^d^	
	*N*	Mean (min, max)	*N*	Mean (min, max)		Estimate (95% CI)	*p* value	Estimate (95% CI)	*p* value
TDLU count^a^	180	17 (0, 186)	545	15 (0, 121)	Multiplicative effect on mean	1.58 (1.30, 1.92)	< 0.001	1.38 (1.15, 1.67)	< 0.001
Adp tissue frac^b^	180	0.53 (0.03, 0.95)	545	0.46 (0.02, 0.95)	Additive effect on mean	− 0.01 (− 0.06, 0.04)	0.66	0.01 (− 0.01, 0.04)	0.27
Mean acini count^a^	177	35 (4, 301)	522	26 (3, 195)	Multiplicative effect on mean	1.66 (1.43, 1.93)	< 0.001	1.60 (1.38, 1.86)	< 0.001
Dilated acini	177	2 (0, 69)	522	1 (0, 10)	Multiplicative effect on mean	1.78 (1.43, 2.23)	< 0.001	1.70 (1.35, 2.15)	< 0.001
Mean avg acini^b^	177	1965 (523, 8455)	522	2718 (544, 12,737)	Additive effect on mean	− 0.40 (− 0.50, − 0.30)	< 0.001	− 0.40 (− 0.50, − 0.30)	< 0.001
Mean cap size^b^	177	9741 (151, 75,885)	522	9009 (37, 73,952)	Additive effect on mean	0.07 (− 0.07, 0.23)	0.34	0.08 (− 0.08, 0.24)	0.32
Mean epi size^b^	177	56,318 (2283, 504,707)	522	52,720 (2457, 327,186)	Additive effect on mean	0.03 (− 0.12, 0.17)	0.73	0.02 (− 0.13, 0.17)	0.80
Mean epi stroma ratio^b^	177	0.37 (0.12, 0.62)	522	0.41 (0.10, 0.95)	Additive effect on mean	− 0.02 (− 0.04, 0.00)	0.13	− 0.01 (− 0.03, 0.01)	0.20
Mononuclear cell count^a^	177	141 (5, 1544)	522	98 (0, 685)	Multiplicative effect on mean	1.54 (1.34, 1.77)	< 0.001	1.51 (1.31, 1.74)	< 0.001
Mean nearby fat^b^	177	161,457 (0, 4,695,569)	522	106,157 (0, 2,920,220)	Additive effect on mean	0.45 (− 3.40, 4.29)	0.82	1.20 (− 2.31, 4.71)	0.50
Mean TDLU size^b^	177	196,285 (25,955, 1,960,288)	522	159,555 (20,508, 1,312,554)	Additive effect on mean	0.11 (− 0.02, 0.25)	0.10	0.10 (− 0.04, 0.24)	0.17

CI = confidence interval; adp = adipose; avg = average; cap = capillary; epi = epithelial; TDLU = terminal duct lobular unit

^a^Negative binomial regression models were used for comparisons between parous and nulliparous women; multiplicative effects on the mean and 95% CIs were estimated and are interpreted as the multiplicative effect on the mean outcome value

^b^Linear regression models were used for comparisons between parous and nulliparous women; additive effects on the mean and 95% CIs were estimated and are interpreted as the additive effect on the mean outcome value (on the natural logarithm scale for mean avg acini, mean cap size, mean epi size, and mean TDLU size, and on the cube root scale for mean nearby fat)

^c^Multivariable models were adjusted for age as well as any variable that differed between parous and nulliparous women with a *p* value < 0.15. *p* values < 0.0045 are considered as statistically significant after applying a Bonferroni correction for multiple testing

^d^P-values < 0.0045 are considered as statistically significant after applying a Bonferroni correction for multiple testing

**Fig. 1:**
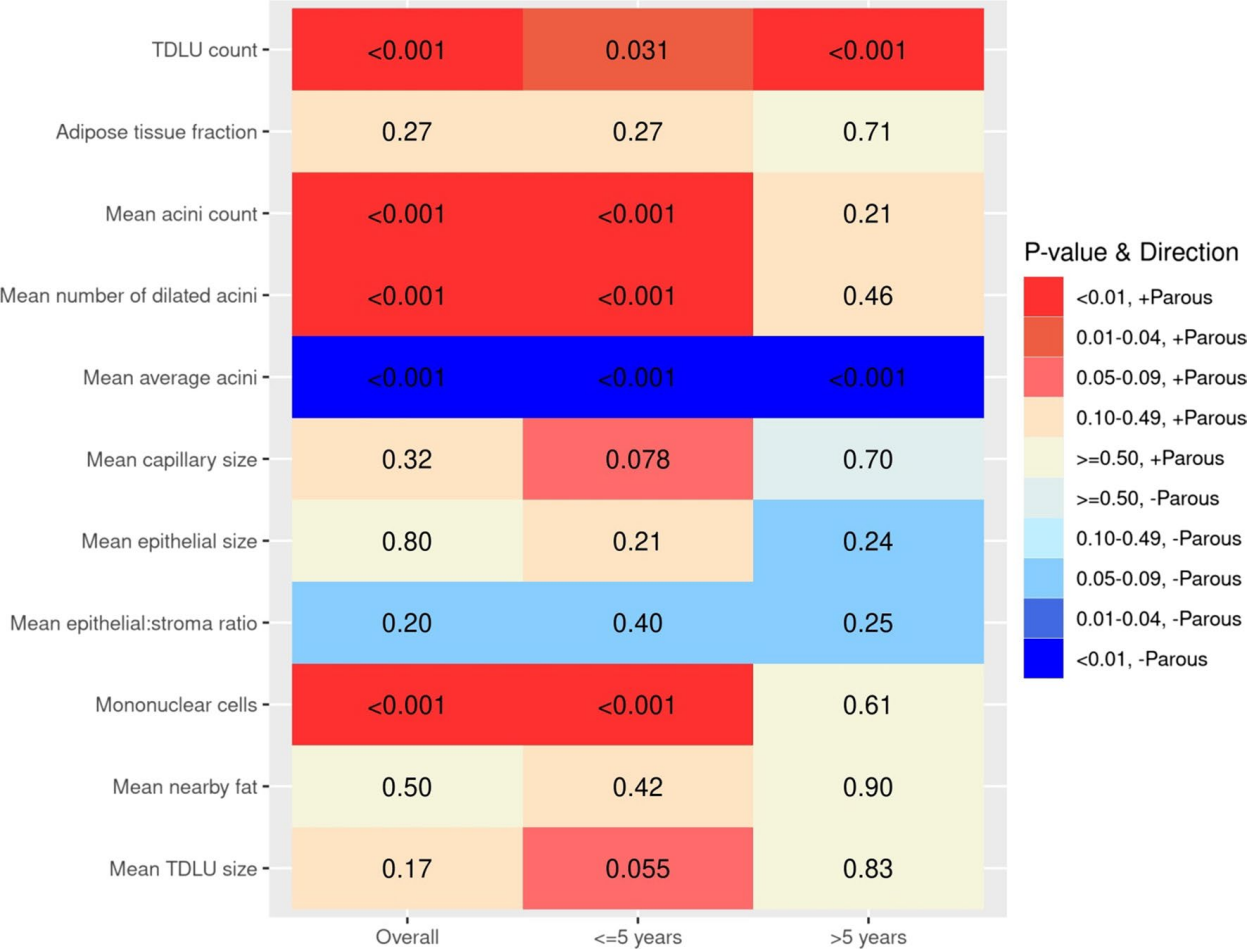
Heat map of p values for pathology Artificial Intelligence analysis data comparing parous women overall, and stratified by ≤ 5 years since birth (*n* = 103) and > 5 years since birth (*n* = 77) versus nulliparas (*n* = 545; referent). *p* values result from negative binomial or linear regression models that were adjusted for age as well as any variable that differed between parous and nulliparous women with a *p* value < 0.15 (race, BMI, percent fat, and relative with breast or ovarian cancer). *p* values < 0.0045 are considered as statistically significant after applying a Bonferroni correction for multiple testing. “ + Parous” means the given outcome measure was higher for parous compared to nulliparous women. “−Parous” means that the given outcome measure was lower for parous compared to nulliparous women

We performed additional analysis stratifying data for parous women as ≤ 5 versus > 5 years postpartum. Irrespective of postpartum interval (i.e., time from most recent birth to donation), AI analysis showed that TDLU counts were higher and acinar area smaller among parous women compared to nulliparous women. In contrast, only specimens obtained within 5 years of childbirth showed higher mean acini counts (multiplicative effect on mean: 1.91, 95% CI = 1.60–2.28; *p* < 0.001), more frequent dilated acini (multiplicative effect on mean: 2.07, 95% CI = 1.59–2.70; *p* < 0.001) and increased mononuclear cells in TDLUs (multiplicative effect on mean: 1.82, 95% CI = 1.54–2.14; *p* < 0.001) compared with those from nulliparas (Fig. 1; Table 3). Similarly, visual analysis confirmed the AI findings above regarding a statistically significant increase in TDLU counts among parous versus nulliparous women, irrespective of the interval from birth to donation, and increased mononuclear cells in TDLUS only in samples obtained within 5 years of a birth (surrogate for all types of immune cells) (Additional file 1: Fig. 2). To assess AI changes evolving over the first 5 years postpartum, we performed an exploratory analysis of AI data for 101 parous women who donated tissue within 5 years of a last birth (per one-year intervals), which revealed suggestive declines in mean acini count (*p* = 0.015), number of dilated acini (*p* = 0.70) and mononuclear cells over the course of the early postpartum period (Additional file 1: table 3: changes per year for years 1–5).

**Table 3:** Comparison of AI data for parous women by time since last birth versus nulliparous women

Outcome	Association measure	Comparison between *N* = 103 parous women with a time since last birth ≤ 5 years and *N* = 545 nulliparous women (reference group)				Comparison between *N* = 77 parous women with a time since last birth > 5 years and *N* = 545 nulliparous women (reference group)			
		Age-adjusted analysis		Adjusting for age, race, BMI, percent fat, and relative with breast or ovarian cancer^c^		Age-adjusted analysis		Adjusting for age, race, BMI, percent fat, and relative with breast or ovarian cancer^c^	
		Estimate (95% CI)	*P* value	Estimate (95% CI)	*p* value	Estimate (95% CI)	*p* value	Estimate (95% CI)	*p* value
TDLU count^a^	Multiplicative effect on mean	1.40 (1.12, 1.76)	0.003	1.27 (1.02, 1.57)	0.031	1.90 (1.45, 2.48)	< 0.001	1.66 (1.28, 2.15)	< 0.001
Adipose tissue frac^b^	Additive effect on mean	− 0.02 (− 0.07, 0.04)	0.52	0.02 (− 0.01, 0.04)	0.27	− 0.00 (− 0.07, 0.06)	0.94	0.01 (− 0.03, 0.04)	0.71
Mean acini count^a^	Multiplicative effect on mean	1.98 (1.67, 2.36)	< 0.001	1.91 (1.60, 2.28)	< 0.001	1.16 (0.95, 1.42)	0.14	1.14 (0.93, 1.41)	0.21
Dilated acini	Multiplicative effect on mean	2.18 (1.70, 2.80)	< 0.001	2.07 (1.59, 2.70)	< 0.001	1.14 (0.87, 1.50)	0.34	1.11 (0.84, 1.48)	0.46
Mean avg acini^b^	Additive effect on mean	− 0.44 (− 0.55, − 0.32)	< 0.001	− 0.46 (− 0.57, − 0.34)	< 0.001	− 0.33 (− 0.47, − 0.19)	< 0.001	− 0.30 (− 0.45, − 0.16)	< 0.001
Mean cap size^b^	Additive effect on mean	0.16 (− 0.02, 0.34)	0.081	0.17 (− 0.02, 0.36)	0.078	− 0.05 (− 0.26, 0.16)	0.62	− 0.04 (− 0.26, 0.18)	0.70
Mean epi size^b^	Additive effect on mean	0.12 (− 0.05, 0.29)	0.16	0.11 (− 0.06, 0.29)	0.21	− 0.12 (− 0.31, 0.07)	0.22	− 0.12 (− 0.32, 0.08)	0.24
Mean epi stroma ratio^b^	Additive effect on mean	− 0.01 (− 0.03, 0.01)	0.41	− 0.01 (− 0.03, 0.01)	0.40	− 0.02 (− 0.05, 0.00)	0.097	− 0.02 (− 0.05, 0.01)	0.25
Mononuclear cell count^a^	Multiplicative effect on mean	1.85 (1.58, 2.18)	< 0.001	1.82 (1.54, 2.14)	< 0.001	1.06 (0.89, 1.27)	0.51	1.05 (0.87, 1.27)	0.61
Mean nearby fat^b^	Additive effect on mean	0.68 (− 3.80, 5.15)	0.77	1.70 (− 2.40, 5.80)	0.42	0.01 (− 5.18, 5.19)	1.00	0.30 (− 4.54, 5.15)	0.90
Mean TDLU size^b^	Additive effect on mean	0.17 (0.01, 0.32)	0.038	0.16 (− 0.00, 0.32)	0.055	0.03 (− 0.15, 0.22)	0.70	0.02 (− 0.17, 0.21)	0.83

CI = confidence interval; adp = adipose; avg = average; cap = capillary; epi = epithelial; TDLU = terminal duct lobular unit

^a^Negative binomial regression models were used for comparisons between parous and nulliparous women; multiplicative effects on the mean and 95% CIs were estimated and are interpreted as the multiplicative effect on the mean outcome value.

^b^Linear regression models were used for comparisons between parous and nulliparous women; additive effects on the mean and 95% CIs were estimated and are interpreted as the additive effect on the mean outcome value (on the natural logarithm scale for mean avg acini, mean cap size, mean epi size, and mean TDLU size, and on the cube root scale for mean nearby fat).

^c^Multivariable models were adjusted for age as well as any variable that differed between parous and nulliparous women with a *P* value < 0.15. *P* values < 0.0045 are considered as statistically significant after applying a Bonferroni correction for multiple testing

### Exploratory analyses of PPI features stratified by parity status and by race

We performed separate multivariable analyses of AI-derived histologic features in nulliparous and in parous tissues. In both groups, higher BMI (and percentage fat in tissue) was associated with statistically significantly lower TDLU counts and older age was related to increased detection of dilated acini (Additional file 1: Tables 4–5). Among parous women, higher numbers of births were related to reduced detection of dilated acini (OR = 0.19; 95%CI: 0.08–0.47; *p* < 0.001) and breastfeeding was related to increased detection of dilated acini (OR = 2.22; 95% CI: 1.30–3.80; *p* < 0.004; Additional file 1: Table 5). Also, among nulliparas, later age at menarche was associated with lower mononuclear cell counts (OR = 0.73, 95% CI: 0.58–0.92; *p* = 0.008; Additional file 1: Table 6). Additional associations that were statistically significant in multivariate analyses but did not reach significance when considering multiple comparisons, included: 1) current ethanol use and increased TDLU numbers in both parous and nulliparous women; increased detection of dilated acini among nulliparas of Hispanic ethnicity and higher mononuclear cell counts among women with a family history of breast/ovarian cancer. To assess effects of breastfeeding after last birth prior to donation (data were collected as total months across all pregnancies), we restricted analyses to uniparous women, which revealed that breastfeeding was associated with reduced TDLU counts (multiplicative effect 0.72, 95%CI = 0.53–0.98; *p* = 0.037); increased frequency of dilated acini (multiplicative effect 2.01, 95%CI = 1.37–2.93; *p* < 0.001), higher mean acini area (additive effect 0.16, 95%CI = 0.01–0.31; *p* = 0.032); increased mean capillary counts (multiplicative effect 1.21, 95%CI = 1.03–1.42; *p* = 0.023) and increased mononuclear cells (multiplicative effect 1.30, 95%CI = 1.08–1.57; *p* = 0.006) (data not shown).

In multivariable analyses, African American women’s tissues showed lower TDLU counts and increased acini counts, capillary area and mononuclear cell counts and TDLU area; however, none of these results were significant after considering multiple comparisons (Additional file 1: Table 7).

### Sensitivity analyses

Given that obesity and percentage of fat on slides are correlated, and that poor correlation may sometimes reflect chance non-representative sampling [8], we re-analyzed data stratified by obesity status, percentage of fat on the slide (≤ 50%; 60–80% and ≥ 90%) and cross-classified by these factors. Results were generally consistent with the analysis presented above (data not shown). Results were substantially unchanged after excluding postmenopausal women.

## Discussion

We report a detailed analysis of the morphologic features of normal nulliparous and parous breast tissues donated for research to the KTB. Important strengths of this analysis include use of AI methods to provide quantitation of morphologic features and inclusion of young women for whom clinical biopsies of normal tissues are rarely available, and among whom effects of PPI are prominent [8]. By defining morphologic characteristics of healthy donated tissues of nulliparous and parous women, we seek to develop a reference for comparison in future studies of BC risk among patients diagnosed with BBD in the postpartum period whose biopsies contain background normal tissue. Future refinement, validation, and testing of AI methods in clinical biopsies may enable development of quantitative reference ranges, which can complement assessment of BBD severity in predicting BC risk.

Our analysis demonstrates that some features that distinguish parous versus nulliparous breast tissues are durable, whereas others are transient. Specifically, we found that sustained higher TDLU counts, as previously shown [8] and reduced acini area per TDLU are features of tissues from parous versus nulliparous women. In contrast, compared with nulliparas, only recently parous women showed higher mean acini counts, mean number of dilated acini and mononuclear cells in TDLUs (including plasma cells) and marginally larger TDLU area. Exploratory analyses of AI data for 101 parous women who donated samples within ≤ 5 years of a birth, suggested that mean acini count, number of dilated acini and number of mononuclear cells declines during this early postpartum interval. These results were generally confirmed by independent visual assessment, although quantitation by AI extends the findings.

Our detection of higher mononuclear cell counts among parous women is consistent with data indicating that RNA expression of inflammation and immune genes is increased after recent childbirth [20–22]. It is hypothesized that the pregnancy-lactation-PPI cycle is linked to distinctive early and late effects; specifically, inflammation immediately postpartum may be associated with higher risk of early onset BCs, whereas differentiation effects that occur later may afford protection against late onset BCs [3–7, 23].

Inflammation is linked to risk of early BC precursors (i.e., BBD) and progression of BBD to BC. BBD risk has been linked to higher circulating pro-inflammatory marker levels [24], and higher urine levels of the metabolites of pro-inflammatory prostaglandins are related to elevated BC risk [25, 26], independent of circulating estrogen levels [27]. Use of anti-inflammatory agents is linked to lower BC risk among BBD patients [28, 29]. In aggregate, these data suggest the hypothesis that dysregulated PPI may result in deleterious inflammation that increases risks of BBD and its progression to BC.

We performed exploratory analyses to define additional BC risk factors that are associated with features of parous and nulliparous breast tissues. Higher BMI (and increased fat in breast tissues) was linked to reduced TDLU numbers, consistent with the inverse associations of premenopausal obesity with both mammographic density (an established BC risk factor) and BC risk. TDLUs are uncommon in breast fat, and when present are highly involuted, providing a plausible explanation for this association. However, associations of BMI and BC risk are complex. Obesity has been linked to elevated risk of triple negative BCs [30], although weight gain was not clearly linked to risk of these BCs in a pooled analysis [31]. Postmenopausal obesity is associated with elevated risk of ER-positive postmenopausal BCs [32]. Ethanol, a risk factor for ER-positive BC [33], was associated with increased TDLU numbers in this analysis. We also noted that number of dilated acini was inversely related to number of livebirths but directly related to breastfeeding, which could have implications for normal TDLU involution or development of non-proliferative BBD with microcalcifications, a frequent target for mammographically-guided biopsy. Analysis of data for last pregnancy preceding tissue donation revealed that breastfeeding was associated with reduced TDLU counts, increased frequency of dilated acini, higher mean acini area, increased mean capillary counts and increased mononuclear cells. Thus, parity and breastfeeding shape normal breast microanatomy, with incompletely defined implications for BC risk.

Based on a limited sample, we found that African American nulliparas have increased TDLU counts, shorter TDLU spans and fewer dilated acini than white nulliparas. Although limited by small sample size, these results are intriguing given that young African American women have a higher incidence of ER-negative BCs and overall higher rates of basal BCs than white women [34]. In accord with other studies (reviewed [35]), we found that African Americans had earlier onset of puberty and a higher BMI than white women, and while we adjusted for these and other factors related to race, residual confounding cannot be excluded. Increases in BMI have temporally paralleled early onset of breast development and menarche, but the interval between these events has widened, which is speculated as contributory to elevated BC risk [35]. Previous studies in KTB reported that African American women had higher circulating levels of insulin-like growth factor binding protein-3 (IGFBP-3) than white women, and that among postmenopausal women of both races, IGFBP-3 was inversely related to TDLU counts [36]. We propose that future studies of normal breast tissues may provide insights into why rates of early onset BCs differ by race.

A meta-analysis of 65 studies found that breastfeeding is associated with reduced BC risk, particularly if exclusive (RR = 0.72, 95%CI: 0.58–0.90) [37]. Data from studies of African American women suggest that limited initiation and duration of breastfeeding may contribute to the high rates of aggressive basal BCs in this group [38, 39]. Although the mechanisms that underlie the protective effect of breastfeeding are unknown, preclinical models suggest that abrupt weaning is associated with increased inflammation, epithelial proliferation, and development of BC [23]. Among singletons in the current analysis, breastfeeding duration was associated with multiple morphologic changes, suggesting that molecular analyses to define protective mechanisms associated with breastfeeding may suggest prevention approaches. However, a notable limitation of human studies is that the effects of pregnancy, lactation and PPI are largely inseparable. For example, we hypothesize that identification of dilated acini after childbirth may reflect milk stasis after weaning; the natural history of such changes is unclear, but development of microcalcifications in dilated “ducts” often leads to biopsies of mammographically detected lesions to rule out BC.

Strengths of our analysis include use of the unique, annotated KTB samples and confirmation of AI data by masked visual assessment. Although sampling is a particular concern in fatty breasts, our sensitivity analyses stratifying by BMI and percent fat in tissues was consistent with our overall results. Our sample sizes were limited for some comparisons, especially for African Americans. Further, a high percentage of women reported a family history of breast or ovarian cancer, which could limit generalizability, although family history was not strongly linked to features of PPI in this analysis or to age-related involution previously [8]. A future study that includes details of each pregnancy and breastfeeding duration may provide additional insights into the pregnancy-lactation cycle and BC risk.

In summary, we report a detailed analysis of normal breast morphology, with a focus on contrasting data for parous versus nulliparous women. Our study identified sustained expansion of TDLU numbers and increased immune responses as characteristic of parous samples, with notable increases in immune responses within five years following childbirth. Further, we show that quantitative characteristics of PPI vary with demographic features and BC risk factors. In future work, we hope to compare data for normal background breast tissues included within BBD biopsies of postpartum women, stratified by BC risk, to the physiological changes described herein to understand the relationship of dysregulated PPI to postpartum BC.

## Supplementary Information

**Additional file 1.** Characteristics of participants by race, results of visual review and subset analyses of AI data.

## Data Availability

The datasets generated during and/or analyzed during the current study are available from the corresponding author or from the Komen Tissue Bank on reasonable request.

## Code availability

Available from authors TDB, JVDL, RAV upon reasonable request.

## References

[1] Wellings SR, Jensen HM, Marcum RG (1975). An atlas of subgross pathology of the human breast with special reference to possible precancerous lesions. J Natl Cancer Inst.

[2] Wellings SR (1980). Development of human breast cancer. Adv Cancer Res.

[3] Wallace TR, Tarullo SE, Crump LS, Lyons TR. Studies of postpartum mammary gland involution reveal novel pro-metastatic mechanisms. J Cancer Metastasis Treat. 2019;5. 10.20517/2394-4722.2019.01 PMC6400586 30847405

[4] Lyons TR, O'Brien J, Borges VF, Conklin MW, Keely PJ, Eliceiri KW (2011). Postpartum mammary gland involution drives progression of ductal carcinoma in situ through collagen and COX-2. Nat Med.

[5] Martinson HA, Jindal S, Durand-Rougely C, Borges VF, Schedin P (2015). Wound healing-like immune program facilitates postpartum mammary gland involution and tumor progression. Int J Cancer.

[6] Jindal S, Gao D, Bell P, Albrektsen G, Edgerton SM, Ambrosone CB (2014). Postpartum breast involution reveals regression of secretory lobules mediated by tissue-remodeling. Breast Cancer Res.

[7] Schedin P, O'Brien J, Rudolph M, Stein T, Borges V (2007). Microenvironment of the involuting mammary gland mediates mammary cancer progression. J Mammary Gland Biol Neoplasia.

[8] Figueroa JD, Pfeiffer RM, Patel DA, Linville L, Brinton LA, Gierach GL, et al. Terminal duct lobular unit involution of the normal breast: implications for breast cancer etiology. J Natl Cancer Inst. 2014;106(10). 10.1093/jnci/dju286 PMC4200067 25274491

[9] Radisky DC, Hartmann LC (2009). Mammary involution and breast cancer risk: transgenic models and clinical studies. J Mammary Gland Biol Neoplasia.

[10] Borges VF, Lyons TR, Germain D, Schedin P (2020). postpartum involution and cancer: an opportunity for targeted breast cancer prevention and treatments?. Cancer Res.

[11] Callihan EB, Gao D, Jindal S, Lyons TR, Manthey E, Edgerton S (2013). Postpartum diagnosis demonstrates a high risk for metastasis and merits an expanded definition of pregnancy-associated breast cancer. Breast Cancer Res Treat.

[12] Goddard ET, Bassale S, Schedin T, Jindal S, Johnston J, Cabral E (2019). Association between postpartum breast cancer diagnosis and metastasis and the clinical features underlying risk. JAMA Netw Open.

[13] Nichols HB, Schoemaker MJ, Cai J, Xu J, Wright LB, Brook MN (2019). Breast cancer risk after recent childbirth: a pooled analysis of 15 prospective studies. Ann Intern Med.

[14] Milanese TR, Hartmann LC, Sellers TA, Frost MH, Vierkant RA, Maloney SD (2006). Age-related lobular involution and risk of breast cancer. J Natl Cancer Inst.

[15] Figueroa JD, Pfeiffer RM, Brinton LA, Palakal MM, Degnim AC, Radisky D (2016). Standardized measures of lobular involution and subsequent breast cancer risk among women with benign breast disease: a nested case-control study. Breast Cancer Res Treat.

[16] Sherman ME, Figueroa JD, Henry JE, Clare SE, Rufenbarger C, Storniolo AM (2012). The Susan G. Komen for the Cure Tissue Bank at the IU Simon Cancer Center: a unique resource for defining the "molecular histology" of the breast. Cancer Prev Res (Phila).

[17] de Bel T, Litjens G, Ogony J, Stallings-Mann M, Carter JM, Hilton T, et al. Automated quantification of levels of breast terminal duct lobular (TDLU) involution using deep learning. NPJ Breast Cancer (In Press). 2022. 10.1038/s41523-021-00378-7 PMC8770616 35046392

[18] van der Laak J, Litjens G, Ciompi F (2021). Deep learning in histopathology: the path to the clinic. Nat Med.

[19] Whyte MB, Kelly P (2018). The normal range: it is not normal and it is not a range. Postgrad Med J.

[20] Asztalos S, Gann PH, Hayes MK, Nonn L, Beam CA, Dai Y (2010). Gene expression patterns in the human breast after pregnancy. Cancer Prev Res (Phila).

[21] Rotunno M, Sun X, Figueroa J, Sherman ME, Garcia-Closas M, Meltzer P (2014). Parity-related molecular signatures and breast cancer subtypes by estrogen receptor status. Breast Cancer Res.

[22] Santucci-Pereira J, Zeleniuch-Jacquotte A, Afanasyeva Y, Zhong H, Slifker M, Peri S (2019). Genomic signature of parity in the breast of premenopausal women. Breast Cancer Res.

[23] Basree MM, Shinde N, Koivisto C, Cuitino M, Kladney R, Zhang J (2019). Abrupt involution induces inflammation, estrogenic signaling, and hyperplasia linking lack of breastfeeding with increased risk of breast cancer. Breast Cancer Res.

[24] Catsburg C, Gunter MJ, Chen C, Cote ML, Kabat GC, Nassir R (2014). Insulin, estrogen, inflammatory markers, and risk of benign proliferative breast disease. Cancer Res.

[25] Kim S, Taylor JA, Milne GL, Sandler DP (2013). Association between urinary prostaglandin E2 metabolite and breast cancer risk: a prospective, case-cohort study of postmenopausal women. Cancer Prev Res (Phila).

[26] Cui Y, Shu XO, Gao YT, Cai Q, Ji BT, Li HL (2014). Urinary prostaglandin E2 metabolite and breast cancer risk. Cancer Epidemiol Biomark Prev.

[27] Kim S, Campbell J, Yoo W, Taylor JA, Sandler DP (2017). Systemic levels of estrogens and PGE2 synthesis in relation to postmenopausal breast cancer risk. Cancer Epidemiol Biomark Prev.

[28] Sherman ME, Vierkant RA, Kaggal S, Hoskin TL, Frost MH, Denison L (2020). Breast cancer risk and use of nonsteroidal anti-inflammatory agents after a benign breast biopsy. Cancer Prev Res (Phila).

[29] Gallicchio L, McSorley MA, Newschaffer CJ, Thuita LW, Huang HY, Hoffman SC (2006). Nonsteroidal antiinflammatory drugs, cyclooxygenase polymorphisms, and the risk of developing breast carcinoma among women with benign breast disease. Cancer.

[30] Pierobon M, Frankenfeld CL (2013). Obesity as a risk factor for triple-negative breast cancers: a systematic review and meta-analysis. Breast Cancer Res Treat.

[31] Premenopausal Breast Cancer Collaborative G, Schoemaker MJ, Nichols HB, Wright LB, Brook MN, Jones ME, et al. Association of Body Mass Index and Age With Subsequent Breast Cancer Risk in Premenopausal Women. JAMA Oncol. 2018;4(11):e181771. 10.1001/jamaoncol.2018.1771 PMC6248078 29931120

[32] Agurs-Collins T, Ross SA, Dunn BK (2019). The many faces of obesity and its influence on breast cancer risk. Front Oncol.

[33] Liu Y, Nguyen N, Colditz GA (2015). Links between alcohol consumption and breast cancer: a look at the evidence. Womens Health (Lond).

[34] Kong X, Liu Z, Cheng R, Sun L, Huang S, Fang Y (2020). Variation in breast cancer subtype incidence and distribution by race/ethnicity in the United States From 2010 to 2015. JAMA Netw Open.

[35] Smith CE, Biro FM (2020). Pubertal development: What's Normal/What's Not. Clin Obstet Gynecol.

[36] Oh H, Pfeiffer RM, Falk RT, Horne HN, Xiang J, Pollak M (2018). Serum insulin-like growth factor (IGF)-I and IGF binding protein-3 in relation to terminal duct lobular unit involution of the normal breast in Caucasian and African American women: the Susan G Komen Tissue Bank. Int J Cancer.

[37] Unar-Munguia M, Torres-Mejia G, Colchero MA, Gonzalez de Cosio T (2017). Breastfeeding Mode and risk of breast cancer: a dose-response meta-analysis. J Hum Lact.

[38] Palmer JR, Viscidi E, Troester MA, Hong CC, Schedin P, Bethea TN, et al. Parity, lactation, and breast cancer subtypes in African American women: results from the AMBER Consortium. J Natl Cancer Inst. 2014;106(10). 10.1093/jnci/dju237 PMC4271113 25224496

[39] Ogony JW, Radisky DC, Ruddy KJ, Goodison S, Wickland DP, Egan KM (2020). Immune responses and risk of triple-negative breast cancer: implications for higher rates among African American Women. Cancer Prev Res (Phila).

