# Supplementary material for: Towards defining morphologic parameters of normal parous and nulliparous breast tissues by artificial intelligence
Source: Breast Cancer Res. 2022 Jul 11;24:45. doi: 10.1186/s13058-022-01541-z (PMC9275035; doi:10.1186/s13058-022-01541-z)
Supplement: Supplementary file 1 — Additional file 1. Characteristics of participants by race, results of visual review and subset analyses of AI data. [file 13058_2022_1541_MOESM1_ESM.docx]

**Supplementary Table 1: Comparisons of characteristics between White women and African American women**

| Variable | N | White women (N=628) | N | African American women (N=97) | P-value |
| --- | --- | --- | --- | --- | --- |
| Age of donation years) | 628 | 27 (18, 45) | 97 | 33 (18, 45) | <0.001 |
| Ethnicity (Hispanic/Latino) | 627 | 49 (7.8%) | 97 | 3 (3.1%) | 0.14 |
| Parity | 628 |  | 97 |  | 0.10 |
| Parous |  | 149 (23.7%) |  | 31 (32.0%) |  |
| Nulliparous |  | 479 (76.3%) |  | 66 (68.0%) |  |
| BMI | 628 | 25.1 (13.3, 63.1) | 97 | 31.6 (19.9, 63.2) | <0.001 |
| Current smoking | 603 | 33 (5.5%) | 93 | 3 (3.2%) | 0.46 |
| Current drinking | 624 | 461 (73.9%) | 97 | 58 (59.8%) | 0.005 |
| Age at first period (years) | 628 | 12 (8, 19) | 97 | 12 (9, 18) | <0.001 |
| Menstrual status | 627 |  | 97 |  | 0.41 |
| Pre-menopausal |  | 604 (96.3%) |  | 94 (96.9%) |  |
| Post-menopausal |  | 12 (1.9%) |  | 3 (3.1%) |  |
| Uterine ablation |  | 11 (1.8%) |  | 0 (0.0%) |  |
| Number of live births | 149 |  | 31 |  | 0.14 |
| 1 |  | 48 (32.2%) |  | 13 (41.9%) |  |
| 2 |  | 74 (49.7%) |  | 9 (29.0%) |  |
| 3 |  | 24 (16.1%) |  | 7 (22.6%) |  |
| 4 |  | 2 (1.3%) |  | 2 (6.5%) |  |
| 5 |  | 1 (0.7%) |  | 0 (0.0%) |  |
| Time since last birth (years) | 149 | 3.7 (0.1, 11.0) | 31 | 6.0 (1.0, 10.9) | 0.012 |
| Age at first birth (years) | 149 | 29 (16, 41) | 31 | 25 (16, 38) | 0.079 |
| History of breastfeeding | 149 | 129 (86.6%) | 31 | 27 (87.1%) | 0.94 |
| Total months breastfeeding | 129 | 9 (1, 90) | 27 | 7 (1, 27) | 0.45 |
| Total months breastfeeding (with no breastfeeding considered to be 0 months) | 149 | 7 (0, 90) | 31 | 6 (0, 27) | 0.57 |
| Any relative with breast/ovarian cancer | 591 | 311 (52.6%) | 88 | 45 (51.1%) | 0.82 |
| The sample median (minimum, maximum) is given for continuous variables. P-values result from a Wilcoxon rank sum test (continuous or ordinal variables) or Fisher’s exact test (categorical variables). | | | | | |

**Supplementary Table 2: Comparison of visual findings between parous and nulliparous women**

|  |  | |  | |  | Comparison between parous women and nulliparous women (reference group) | | | |
| --- | --- | --- | --- | --- | --- | --- | --- | --- | --- |
|  | Parous women (N=180) | | Nulliparous women (N=545) | |  | Age-adjusted analysis | | Adjusting for age, race, BMI, percent fat, and relative with breast or ovarian cancer^5^ | |
| Outcome | N | Median (min, max) or No. (%) | N | Median (min, max) or No. (%) | Association measure | Estimate (95% CI) | P-value | Estimate (95% CI) | P-value |
| TDLU number^1^ | 180 | 20 (0, 190) | 545 | 14 (0, 123) | Multiplicative effect on mean | 1.86 (1.53, 2.27) | <0.001 | 1.70 (1.40, 2.07) | <0.001 |
| Acini number^2^ | 173 | 1.5 (1.0, 4.7) | 520 | 1.7 (1.0, 6.0) | Odds ratio | 1.15 (0.80, 1.65) | 0.44 | 1.04 (0.72, 1.52) | 0.83 |
| TDLU Span (um)^3^ | 173 | 286 (124, 985) | 520 | 331 (108, 2084) | Multiplicative effect on median | 0.96 (0.89, 1.04) | 0.38 | 0.94 (0.86, 1.02) | 0.13 |
| BBD^4^ | 139 | 34 (24.5%) | 373 | 62 (16.6%) | Odds ratio | 1.00 (0.57, 1.74) | 1.00 | 0.84 (0.45, 1.55) | 0.58 |
| Plasma cells^4^ | 147 | 90 (61.2%) | 381 | 188 (49.3%) | Odds ratio | 1.33 (0.84, 2.10) | 0.23 | 1.38 (0.85, 2.23) | 0.20 |
| Inflammation^4^ | 147 | 37 (25.2%) | 381 | 57 (15.0%) | Odds ratio | 1.46 (0.84, 2.54) | 0.18 | 1.54 (0.87, 2.72) | 0.14 |
| TDLU = terminal duct lobular unit. CI=confidence interval. ^1^ For comparisons of TDLU number, negative binomial regression models were used; multiplicative effects on the mean and 95% confidence intervals (CIs) were estimated and are interpreted as the multiplicative effect on the mean TDLU number. In descriptive summaries, the mean TDLU number was given rather than the median, in order to correspond to the multiplicative effects on the mean that result from negative binomial regression models. ^2^ For comparisons of acini number, the mean acini number per patient was evaluated, and proportional odds logistic regression models were used; odds ratios (ORs) and 95% CIs were estimated and are interpreted as the multiplicative increase in the odds of a higher acini number category (1.00, 1.01-2.00, 2.01-3.00, 3.01-4.00, >4). ^3^ For comparisons of span, the mean span per patient was evaluated, and linear regression models were used where span was examined on the natural logarithm scale owing to its skewed distribution. Multiplicative effects on the median and 95% CIs were estimated; these multiplicative effects were obtained by exponentiating regression coefficients. ^4^ For comparisons of BBD, plasma cells, and inflammation, binary logistic regression models were used; ORs and 95% CIs were estimated and are interpreted as the multiplicative increase in the odds of the given outcome. ^5^ Multivariable models were adjusted for age as well as any variable that differed between parous and nulliparous women with a p-value <0.15. P-values <0.0083 are considered as statistically significant after applying a Bonferroni correction for multiple testing. | | | | | | | | | |

**Supplementary Table 3. Associations of length of time since last birth and AI outcomes in the 101 parous women with a time since last birth ≤ 5 years**

|  |  |  | Association between time since last birth (as a continuous variable, per each 1-year increase) and the given outcome | | | |
| --- | --- | --- | --- | --- | --- | --- |
|  |  |  | Unadjusted analysis | | Adjusting for age | |
| Outcome | N | Association measure | Estimate (95% CI) | P-value | Estimate (95% CI) | P-value |
| TDLU count^1^ | 101 | Multiplicative effect on mean | 0.97 (0.83, 1.13) | 0.71 | 0.97 (0.83, 1.13) | 0.71 |
| Adp tissue frac^2^ | 101 | Additive effect on mean | 0.04 (-0.00, 0.08) | 0.061 | 0.04 (-0.00, 0.08) | 0.069 |
| Mean acini count^1^ | 99 | Multiplicative effect on mean | 0.85 (0.75, 0.97) | 0.016 | 0.85 (0.75, 0.97) | 0.015 |
| S/O BBD | 99 | Multiplicative effect on mean | 0.70 (0.58, 0.85) | <0.001 | 0.70 (0.58, 0.85) | <0.001 |
| Mean avg acini^2^ | 99 | Additive effect on mean | 0.07 (0.00, 0.15) | 0.047 | 0.07 (-0.00, 0.14) | 0.060 |
| Mean cap size^2^ | 99 | Additive effect on mean | -0.06 (-0.21, 0.08) | 0.38 | -0.07 (-0.21, 0.08) | 0.37 |
| Mean epi size^2^ | 99 | Additive effect on mean | -0.08 (-0.22, 0.06) | 0.28 | -0.08 (-0.22, 0.06) | 0.27 |
| Mean epi stroma ratio^2^ | 99 | Additive effect on mean | -0.01 (-0.02, 0.01) | 0.40 | -0.01 (-0.02, 0.01) | 0.40 |
| Mononuclear cell count^1^ | 99 | Multiplicative effect on mean | 0.88 (0.77, 1.00) | 0.058 | 0.88 (0.77, 1.00) | 0.052 |
| Mean nearby fat^2^ | 99 | Additive effect on mean | 0.35 (-3.58, 4.28) | 0.86 | 0.16 (-3.77, 4.09) | 0.94 |
| Mean TDLU size^2^ | 99 | Additive effect on mean | -0.06 (-0.20, 0.07) | 0.36 | -0.06 (-0.20, 0.07) | 0.35 |
| CI=confidence interval; adp=adipose; avg=average; cap=capillary; epi=epithelial; TDLU=terminal duct lobular unit. S/O BBD refers to dilated acini suggestive of benign breast disease. ^1^ Negative binomial regression models were used; multiplicative effects on the mean and 95% CIs were estimated and are interpreted as the multiplicative effect on the mean outcome value for each 1-year increase in time since last birth. ^2^ Linear regression models were used; additive effects on the mean and 95% CIs were estimated and are interpreted as the additive effect on the mean outcome value (on the natural logarithm scale for mean avg acini, mean cap size, mean epi size, and mean TDLU size, and on the cube root scale for mean nearby fat) for each 1-year increase in time since last birth. P-values <0.0045 are considered as statistically significant after applying a Bonferroni correction for multiple testing. | | | | | | |

**Supplementary Table 4: Associations between patient characteristics and TDLU number assessed by AI separately for parous and nulliparous women**

|  | Parous women (N=180) | | | | Nulliparous women (N=545) | | | |
| --- | --- | --- | --- | --- | --- | --- | --- | --- |
|  | Unadjusted analysis | | Adjusting for age of donation, BMI, percent fat, and current drinking | | Unadjusted analysis | | Adjusting for age of donation, BMI, percent fat, and current smoking | |
| Variable | Multiplicative effect on mean (95% CI) | P-value | Multiplicative effect on mean (95% CI) | P-value | Multiplicative effect on mean (95% CI) | P-value | Multiplicative effect on mean (95% CI) | P-value |
| Age of donation (5 year increase) | 0.95 (0.81, 1.11) | 0.51 | 1.02 (0.90, 1.16) | 0.71 | 0.85 (0.81, 0.91) | <0.001 | 1.04 (0.91, 1.20) | 0.53 |
| Ethnicity (Hispanic/Latino) | 1.03 (0.58, 1.84) | 0.91 | 0.93 (0.57, 1.53) | 0.79 | 0.91 (0.65, 1.28) | 0.59 | 0.95 (0.54, 1.69) | 0.86 |
| BMI (5 unit increase) | 0.87 (0.80, 0.96) | 0.004 | 0.87 (0.80, 0.96) | 0.003 | 0.92 (0.87, 0.97) | 0.002 | 0.88 (0.80, 0.97) | 0.009 |
| Percent fat (10 unit increase) | 0.80 (0.76, 0.84) | <0.001 | 0.80 (0.77, 0.84) | <0.001 | 0.85 (0.83, 0.88) | <0.001 | 0.80 (0.76, 0.85) | <0.001 |
| Current smoking | 0.51 (0.20, 1.27) | 0.15 | 0.62 (0.29, 1.33) | 0.22 | 0.62 (0.43, 0.90) | 0.013 | 0.68 (0.31, 1.46) | 0.32 |
| Current drinking | 1.53 (1.11, 2.11) | 0.010 | 1.32 (1.00, 1.74) | 0.048 | 0.89 (0.73, 1.08) | 0.24 | 1.35 (1.02, 1.80) | 0.037 |
| Age at first period (1 year increase) | 1.01 (0.90, 1.12) | 0.90 | 0.94 (0.87, 1.03) | 0.17 | 0.98 (0.91, 1.04) | 0.46 | 0.94 (0.86, 1.02) | 0.14 |
| Menstrual status (Post-menopausal or uterine ablation) | 0.62 (0.29, 1.35) | 0.23 | 0.71 (0.37, 1.36) | 0.30 | 0.76 (0.47, 1.22) | 0.26 | 0.70 (0.36, 1.35) | 0.28 |
| Number of live births (1 unit increase) | 0.88 (0.71, 1.09) | 0.25 | 1.01 (0.85, 1.21) | 0.89 | N/A | N/A | N/A | N/A |
| Time since last birth (1 year increase) | 0.98 (0.93, 1.03) | 0.40 | 1.00 (0.95, 1.05) | 0.94 | N/A | N/A | N/A | N/A |
| Age at first birth (5 year increase) | 1.09 (0.93, 1.28) | 0.28 | 1.09 (0.93, 1.27) | 0.31 | N/A | N/A | N/A | N/A |
| History of breastfeeding | 0.79 (0.51, 1.22) | 0.29 | 0.96 (0.66, 1.38) | 0.81 | N/A | N/A | N/A | N/A |
| Total months breastfeeding (12 month increase)^1^ | 0.95 (0.82, 1.12) | 0.55 | 0.97 (0.85, 1.11) | 0.67 | N/A | N/A | N/A | N/A |
| Relative with breast/ovarian cancer | 1.25 (0.91, 1.71) | 0.17 | 1.11 (0.85, 1.44) | 0.45 | 1.06 (0.89, 1.27) | 0.53 | 1.09 (0.83, 1.44) | 0.54 |
| BMI= body mass index; Percent fat=area of fat / total area of tissue X 100; CI=confidence interval. Multiplicative effects, 95% CIs, and p-values result from negative binomial regression models. Multivariable models were adjusted for age of donation (as a continuous variable) as well as any variable that had a p-value <0.05 in unadjusted analysis. To avoid collinearity, models for BMI were never adjusted for percent fat, and conversely models for percent fat were never adjusted for BMI. Multiplicative effects are interpreted as the multiplicative effect on the mean TDLU number. P-values <0.0036 (parous women) and <0.0056 (nulliparous women) are considered as statistically significant after applying a Bonferroni correction for multiple testing. ^1^ Women who had no history of breastfeeding were considered to have a total months breastfeeding value equal to zero. | | | | | | | | |

**Supplementary Table 5: Associations between patient characteristics and dilated acini separately for parous and nulliparous women**

|  | Parous women (N=180) | | | | Nulliparous women (N=545) | | | |
| --- | --- | --- | --- | --- | --- | --- | --- | --- |
|  | Unadjusted analysis | | Adjusting for age of donation percent fat, number of live births, and total months breastfeeding | | Unadjusted analysis | | Adjusting for age of donation and ethnicity | |
| Variable | OR (95% CI) | P-value | OR (95% CI) | P-value | OR (95% CI) | P-value | OR (95% CI) | P-value |
| Age of donation (5 year increase) | 1.44 (0.95, 2.160 | 0.083 | 2.10 (1.25, 3.54) | 0.005 | 1.28 (1.07, 1.54) | 0.008 | 1.27 (1.05, 1.52) | 0.012 |
| Ethnicity (Hispanic/Latino) | 1.87 (0.51, 6.81) | 0.34 | 0.76 (0.16, 3.73) | 0.74 | 3.12 (1.31, 7.43) | 0.010 | 2.91 (1.21, 7.03) | 0.017 |
| BMI (5 unit increase) | 0.79 (0.58, 1.07) | 0.13 | 0.72 (0.50, 1.05) | 0.086 | 0.87 (0.70, 1.07) | 0.18 | 0.75 (0.60, 0.95) | 0.018 |
| Percent fat (10 unit increase) | 0.85 (0.73, 0.99) | 0.035 | 0.81 (0.68, 0.97) | 0.022 | 0.94 (0.85, 1.04) | 0.24 | 0.90 (0.81, 1.01) | 0.065 |
| Current smoking^1^ | N/A | 0.57 | N/A | N/A | 1.98 (0.69, 5.73) | 0.21 | 1.77 (0.59, 5.29) | 0.30 |
| Current drinking | 1.20 (0.49, 2.95) | 0.70 | 1.11 (0.40, 3.07) | 0.84 | 0.85 (0.47, 1.55) | 0.60 | 0.75 (0.41, 1.39) | 0.36 |
| Age at first period (1 year increase) | 1.03 (0.79, 1.34) | 0.85 | 1.09 (0.77, 1.56) | 0.63 | 0.96 (0.78, 1.17) | 0.67 | 0.99 (0.80, 1.21) | 0.88 |
| Menstrual status (Post-menopausal or uterine ablation) | 1.25 (0.23, 6.76) | 0.80 | 2.72 (0.37, 20.01) | 0.33 | 0.55 (0.07, 4.41) | 0.57 | 0.48 (0.06, 3.89) | 0.49 |
| Number of live births (1 unit increase) | 0.49 (0.27, 0.89) | 0.018 | 0.19 (0.08, 0.47) | <0.001 | N/A | N/A | N/A | N/A |
| Time since last birth (1 year increase) | 1.12 (0.99, 1.27) | 0.078 | 1.15 (0.98, 1.36) | 0.091 | N/A | N/A | N/A | N/A |
| Age at first birth (5 year increase) | 1.33 (0.89, 1.99) | 0.17 | 0.51 (0.25, 1.03) | 0.062 | N/A | N/A | N/A | N/A |
| History of breastfeeding | 0.82 (0.27, 2.49) | 0.73 | 0.53 (0.13, 2.18) | 0.37 | N/A | N/A | N/A | N/A |
| Total months breastfeeding (12 month increase)^1^ | 1.49 (1.01, 2.18) | 0.043 | 2.22 (1.30, 3.80) | 0.004 | N/A | N/A | N/A | N/A |
| Relative with breast/ovarian cancer | 0.97 (0.42, 2.27) | 0.95 | 0.89 (0.33, 2.37) | 0.82 | 0.95 (0.54, 1.67) | 0.85 | 0.92 (0.51, 1.65) | 0.78 |
| BMI= body mass index; Percent fat=area of fat / total area of tissue X 100; OR=odds ratio; CI=confidence interval. ORs, 95% CIs, and p-values result from binary logistic regression models. Multivariable models were adjusted for age of donation (as a continuous variable) as well as any variable that had a p-value <0.05 in unadjusted analysis. To avoid collinearity, models for BMI were never adjusted for percent fat, and conversely models for percent fat were never adjusted for BMI. ORs are interpreted as the multiplicative effect on odds of BBD. ^1^ Logistic regression analysis was not possible for current smoking in the parous women owing to the presence of a zero cell count; the p-value in the unadjusted analysis results from Fisher’s exact test. P-values <0.0036 (parous women) and <0.0056 (nulliparous women) are considered as statistically significant after applying a Bonferroni correction for multiple testing. ^1^ Women who had no history of breastfeeding were considered to have a total months breastfeeding value equal to zero. | | | | | | | | |

**Supplementary Table 6: Associations between patient characteristics and AI detection of mononuclear cells separately for parous and nulliparous women**

|  | Parous women (N=180) | | | | Nulliparous women (N=545) | | | |
| --- | --- | --- | --- | --- | --- | --- | --- | --- |
|  | Unadjusted analysis | | Adjusting for age of donation, percent fat, time since last birth, and relative with breast/ovarian cancer | | Unadjusted analysis | | Adjusting for age of donation, BMI, and age at first period | |
| Variable | OR (95% CI) | P-value | OR (95% CI) | P-value | OR (95% CI) | P-value | OR (95% CI) | P-value |
| Age of donation (5 year increase) | 1.00 (0.69, 1.45) | 0.99 | 1.41 (0.90, 2.23) | 0.14 | 1.21 (1.00, 1.46) | 0.050 | 1.14 (0.93, 1.40) | 0.22 |
| Ethnicity (Hispanic/Latino) | 0.64 (0.13, 3.11) | 0.58 | 0.59 (0.11, 3.12) | 0.54 | 2.24 (0.90, 5.60) | 0.085 | 2.10 (0.82, 5.43) | 0.12 |
| BMI (5 unit increase) | 0.77 (0.57, 1.03) | 0.081 | 0.82 (0.59, 1.12) | 0.21 | 1.24 (1.04, 1.47) | 0.019 | 1.10 (0.90, 1.34) | 0.37 |
| Percent fat (10 unit increase) | 0.84 (0.73, 0.97) | 0.021 | 0.86 (0.73, 1.00) | 0.055 | 1.03 (0.92, 1.15) | 0.62 | 1.00 (0.89, 1.12) | 0.96 |
| Current smoking | 1.06 (0.11, 10.58) | 0.96 | 1.12 (0.08, 15.37) | 0.93 | 1.09 (0.31, 3.88) | 0.89 | 0.84 (0.22, 3.12) | 0.79 |
| Current drinking | 2.34 (0.89, 6.14) | 0.083 | 2.11 (0.75, 5.92) | 0.15 | 1.26 (0.66, 2.41) | 0.49 | 1.32 (0.68, 2.59) | 0.42 |
| Age at first period (1 year increase) | 1.10 (0.85, 1.42) | 0.48 | 1.18 (0.87, 1.60) | 0.28 | 0.70 (0.56, 0.87) | 0.001 | 0.73 (0.58, 0.92) | 0.008 |
| Menstrual status (Post-menopausal or uterine ablation)^1^ | N/A | 0.19 | N/A | N/A | 1.30 (0.27, 6.16) | 0.74 | 0.96 (0.20, 4.68) | 0.95 |
| Number of live births (1 unit increase) | 0.91 (0.55, 1.50) | 0.71 | 0.99 (0.54, 1.79) | 0.96 | N/A | N/A | N/A | N/A |
| Time since last birth (1 year increase) | 0.87 (0.76, 0.99) | 0.036 | 0.86 (0.74, 1.00) | 0.047 | N/A | N/A | N/A | N/A |
| Age at first birth (5 year increase) | 1.32 (0.90, 1.94) | 0.16 | 1.20 (0.61, 2.35) | 0.60 | N/A | N/A | N/A | N/A |
| History of breastfeeding | 1.40 (0.44, 4.50) | 0.57 | 1.24 (0.35, 4.34) | 0.74 | N/A | N/A | N/A | N/A |
| Total months breastfeeding (12 month increase)^1^ | 0.97 (0.67, 1.42) | 0.89 | 0.90 (0.60, 1.35) | 0.61 | N/A | N/A | N/A | N/A |
| Relative with breast/ovarian cancer | 3.18 (1.28, 7.91) | 0.013 | 2.91 (1.13, 7.50) | 0.027 | 0.96 (0.53, 1.72) | 0.89 | 0.91 (0.50, 1.65) | 0.75 |
| BMI= body mass index; Percent fat=area of fat / total area of tissue X 100; OR=odds ratio; CI=confidence interval. ORs, 95% CIs, and p-values result from binary logistic regression models. Multivariable models were adjusted for age of donation (as a continuous variable) as well as any variable that had a p-value <0.05 in unadjusted analysis. To avoid collinearity, models for BMI were never adjusted for percent fat, and conversely models for percent fat were never adjusted for BMI. ORs are interpreted as the multiplicative effect on odds of inflammation. ^1^ Logistic regression analysis was not possible for menstrual status in the parous women owing to the presence of a zero cell count; the p-value in the unadjusted analysis results from Fisher’s exact test. P-values <0.0036 (parous women) and <0.0056 (nulliparous women) are considered as statistically significant after applying a Bonferroni correction for multiple testing. ^1^ Women who had no history of breastfeeding were considered to have a total months breastfeeding value equal to zero. | | | | | | | | |

**Supplementary Table 7: Comparison of outcomes between African American women and white women**

|  |  | |  | |  | Comparison between African American and white women (reference group) | | | |
| --- | --- | --- | --- | --- | --- | --- | --- | --- | --- |
|  | African American women (N=97) | | White women (N=628) | |  | Age-adjusted analysis | | Adjusting for age, ethnicity, parity, BMI, percent fat, current drinking, and age at first period^3^ | |
| Outcome | N | Mean (min, max) | N | Mean (min, max) | Association measure | Estimate (95% CI) | P-value | Estimate (95% CI) | P-value |
| TDLU count^1^ | 97 | 11 (0, 81) | 628 | 16 (0, 186) | Multiplicative effect on mean | 0.69 (0.55, 0.86) | 0.001 | 0.77 (0.63, 0.95) | 0.014 |
| Adipose tissue fraction^2^ | 97 | 0.55 (0.03, 0.95) | 628 | 0.47 (0.02, 0.95) | Additive effect on mean | 0.05 (-0.01, 0.10) | 0.083 | -0.00 (-0.03, 0.02) | 0.82 |
| Mean acini count^1^ | 90 | 32 (5, 167) | 609 | 28 (3, 301) | Multiplicative effect on mean | 1.19 (1.00, 1.42) | 0.048 | 1.31 (1.10, 1.57) | 0.003 |
| Mean dilated acini ^1^ | 90 | 2 (0, 25) | 609 | 2 (0, 69) | Multiplicative effect on mean | 1.01 (0.78, 1.33) | 0.90 | 1.02 (0.77, 1.35) | 0.90 |
| Mean average acini^2^ | 90 | 2381 (807, 8455) | 609 | 2549 (523, 12737) | Additive effect on mean | -0.05 (-0.17, 0.07) | 0.41 | -0.09 (-0.20, 0.03) | 0.14 |
| Mean capillary size^2^ | 90 | 10241 (1592, 48946) | 609 | 9039 (37, 75885) | Additive effect on mean | 0.19 (0.02, 0.37) | 0.032 | 0.20 (0.02, 0.38) | 0.033 |
| Mean epithelial size^2^ | 90 | 60033 (5860, 504707) | 609 | 52685 (2283, 418371) | Additive effect on mean | 0.15 (-0.02, 0.31) | 0.082 | 0.17 (-0.00, 0.34) | 0.053 |
| Mean epithelial stroma ratio^2^ | 90 | 0.38 (0.12, 0.69) | 609 | 0.40 (0.10, 0.95) | Additive effect on mean | -0.01 (-0.03, 0.02) | 0.62 | -0.00 (-0.03, 0.02) | 0.74 |
| Mean mononuclear cell count^1^ | 90 | 128 (12, 583) | 609 | 106 (0, 1544) | Multiplicative effect on mean | 1.19 (1.02, 1.40) | 0.032 | 1.24 (1.05, 1.46) | 0.011 |
| Mean nearby fat^2^ | 90 | 152348 (0, 796284) | 609 | 115403 (0, 4695569) | Additive effect on mean | 4.37 (-0.07, 8.80) | 0.054 | 2.96 (-1.27, 7.20) | 0.17 |
| Mean TDLU size^2^ | 90 | 191871 (20508, 1119866) | 609 | 165455 (23764, 1960288) | Additive effect on mean | 0.15 (-0.00, 0.31) | 0.054 | 0.17 (0.01, 0.34) | 0.035 |
| TDLU-terminal duct lobular unit; S/O BBD refers to dilated acini suggestive of benign breast disease; CI=confidence interval. ^1^ Negative binomial regression models were used for comparisons between parous and nulliparous women; multiplicative effects on the mean and 95% CIs were estimated and are interpreted as the multiplicative effect on the mean outcome value. ^2^ Linear regression models were used for comparisons between parous and nulliparous women; additive effects on the mean and 95% CIs were estimated and are interpreted as the additive effect on the mean outcome value (on the natural logarithm scale for epi frac, mean Avg acini, mean Cap size, mean Epi size, and mean TDLU size, and on the cube root scale for TDLU frac and mean Nearby fat). ^3^ Multivariable models were adjusted for age as well as any variable that differed between White and African American women with a p-value <0.15. P-values <0.0045 are considered as statistically significant after applying a Bonferroni correction for multiple testing. | | | | | | | | | |

**Supplemental Methods**

In this section we describe our method for automatically extracting quantitative measures. As a first step, tissue locations were automatically discerned from background in all whole slide images (WSIs) using a background segmentation convolutional neural network (Bandi). Second, within all tissue areas, we apply a structure delineation network for breast tissue described elsewhere (citation will follow). A bird’s eye view of the segmentation is shown in Figure 1.Slide level features considered in this study were limited to: TDLU count, and adipose tissue area, based on pixels. TDLU tissue area included the intralobular stroma, epithelium and epithelial border classes. The TDLU candidates were automatically determined by extracting all areas of intralobular stroma. Subsequently, candidates were excluded if less than three epithelial components – an area of pixels classified as epithelium surrounded by a border - were present. The remaining candidates were considered TDLU detections and included in the TDLU count.For each TDLU detected, we measured the following: acini count, dilated acini count, average acini area, small vessel “capillary” area, epithelial area, epithelium/stroma ratio (ratio of pixels), mononuclear cell count, nearby adipose tissue area, TDLU area. All area measures were calculated by counting the associated pixels, i.e. we counted all pixels classified as vessel for the small vessel area measurement. The approximation of the mononuclear cell count per TDLU is demonstrated in Figure 3. An example of a TDLU candidate with its associated measurements is shown in Figure 4.

All analysis were performed using the following Python (3.6) packages: scikit-learn (0.20.3), NumPy (1.16.2), pandas (0.24.2), SciPy (1.2.1).

**References**

Bándi, Péter, et al. "Resolution-agnostic tissue segmentation in whole-slide histopathology images with convolutional neural networks." *PeerJ* 7 (2019): e8242.

**Supplementary Figure 1.** Quantitation of breast tissue features related to scanned whole slide images (Aperio ScanScope XT Slide Scanner, Leica Biosystems) at 20X with resolution of 0.495 X 0.495 μm^2^ per pixel). Panel A: bird’s eye view of a slide before and after tissue background separation and structure delineation. Panel B: TDLU candidates are automatically extracted from the segmentation by grouping together all epithelial components within an area of intralobular stroma. Candidates are then excluded if there are less than three epithelial components present. The above picture demonstrates the extraction of three TDLUs from a small piece of tissue. Panel C: Breakdown of the extra epithelial cell count extraction for a single TDLU (a). First, the segmentation of the TDLU (b) is used to extract the intralobular stromal area of the TDLU (c). Individual cells were extracted from the stromal area, excluding cells with pixel area smaller than 25, an eccentricity greater than 0.85 and solidity smaller than 0.7 (d). Panel D: Example of a TDLU candidate detection and its associated measurements.

**Supplementary Figure 2**. Forest plot showing comparisons of outcomes between parous and nulliparous women, where parous women were stratified according to time since last birth (≤5 years or >5 years). Effect sizes are in comparison to the nulliparous group and correspond to the multiplicative effect on the mean (TDLU number), the odds ratio for a higher acini number category (acini number), the multiplicative effect on the median (span), and the odds ratio for presence of the given outcome (BBD, plasma cells, inflammation). Effect sizes and 95% confidence intervals (CIs) result from multivariable regression models that were adjusted for age, race, BMI, percent fat, and relative with breast or ovarian cancer. ** Indicates a significant finding after applying a Bonferroni correction for multiple testing (P<0.0083). * Indicates a nominally significant finding (P<0.05) that did not survive Bonferroni correction for multiple testing.

Supplementary Figure 1A


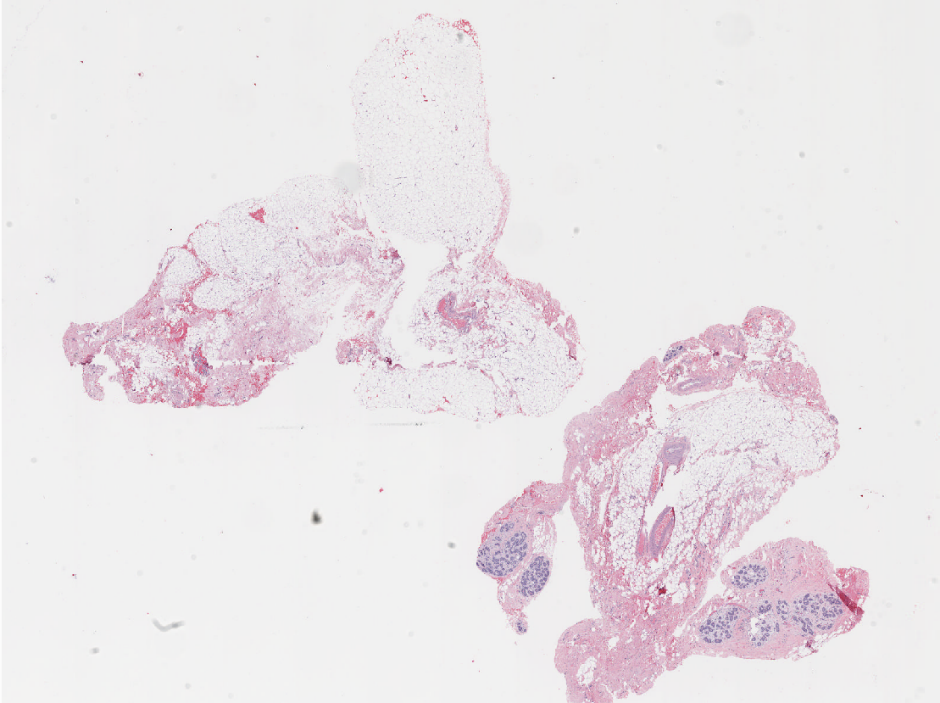

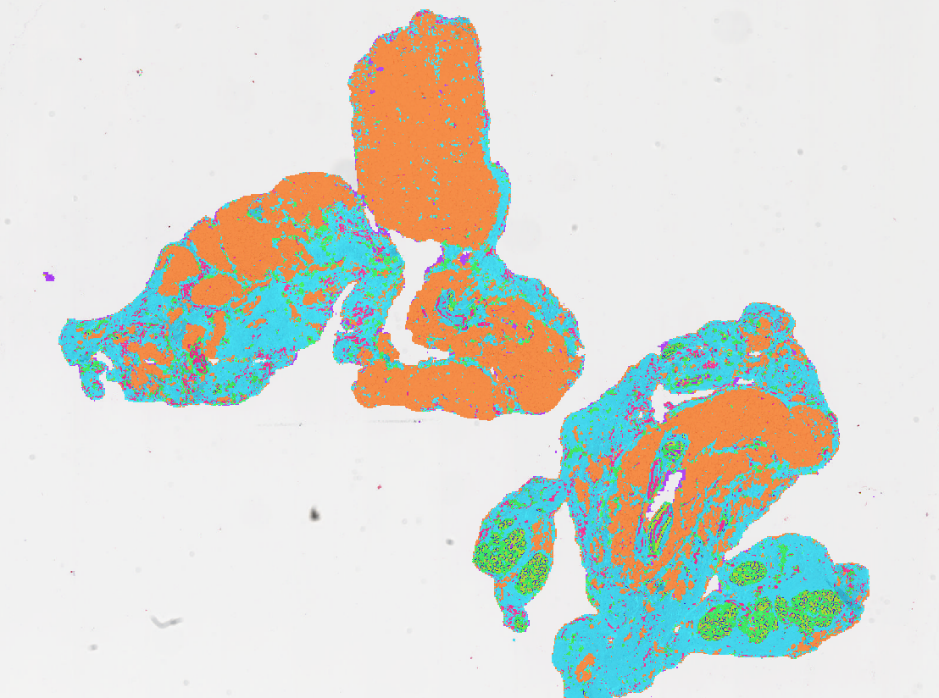


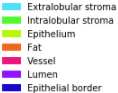


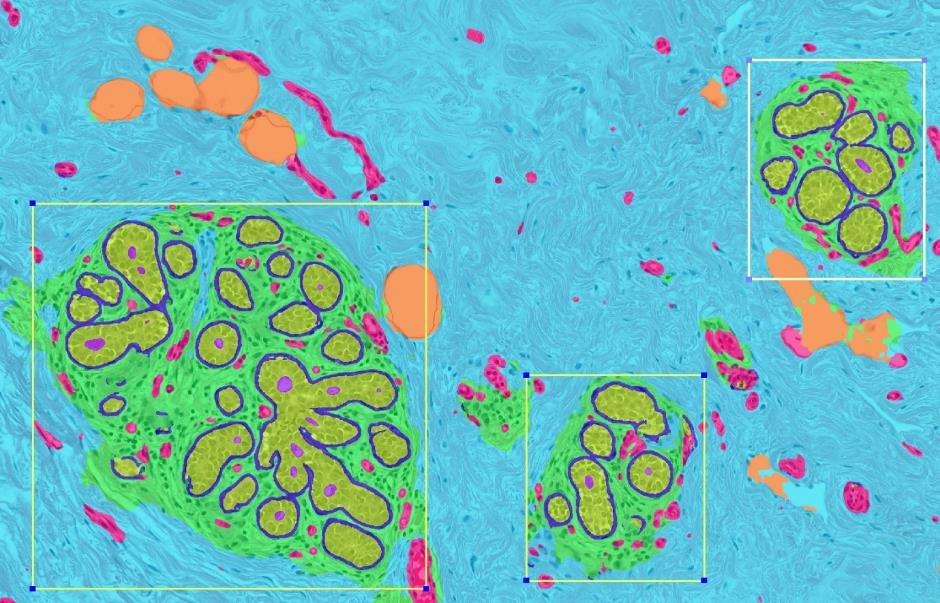

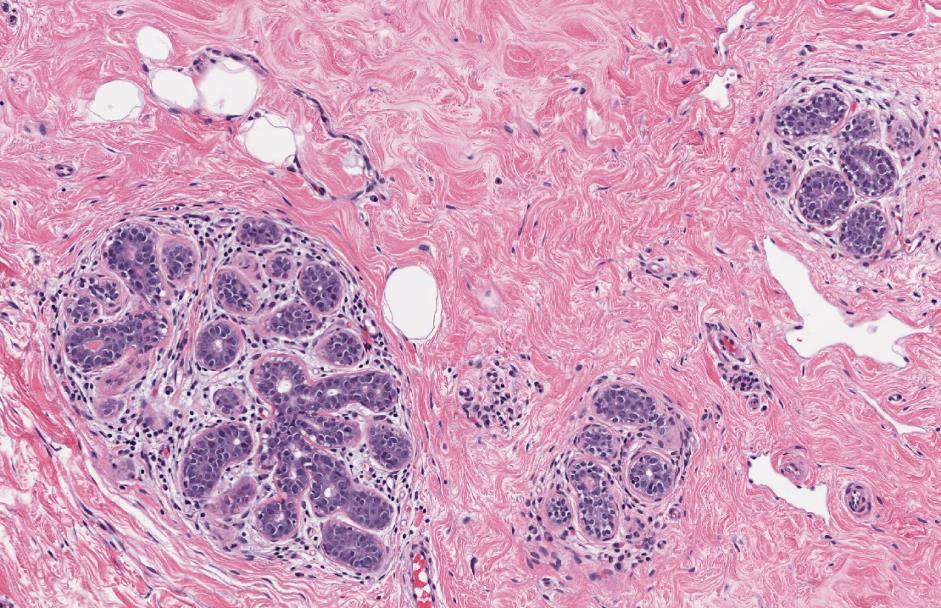


Supplementary Figure 1B.

Supplementary Figure 1C.


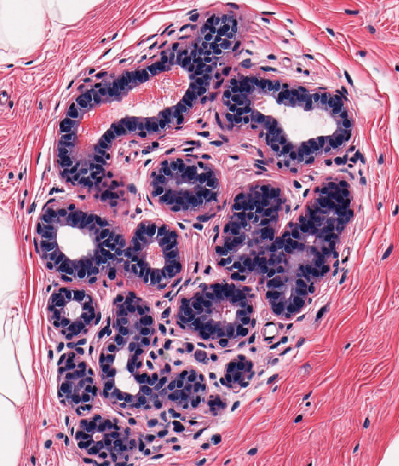

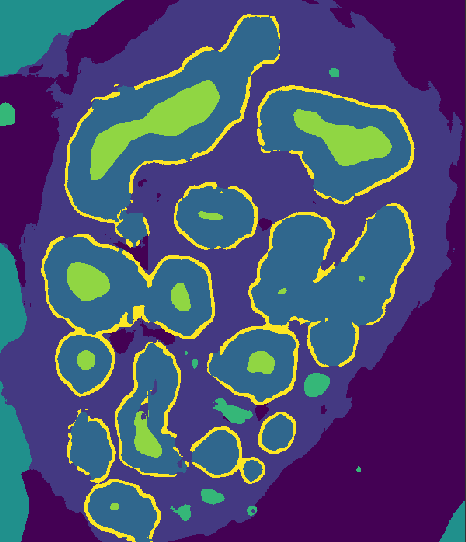


(a)

(b)


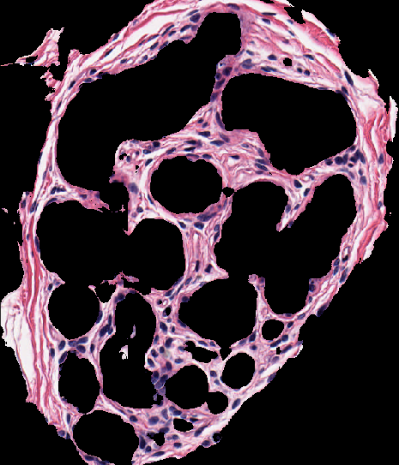


(c)


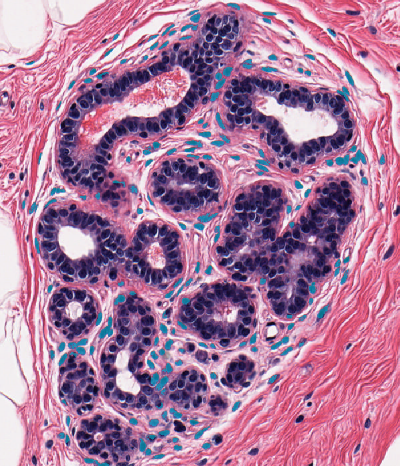


(d)

Supplementary Figure 1D.


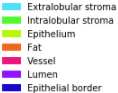

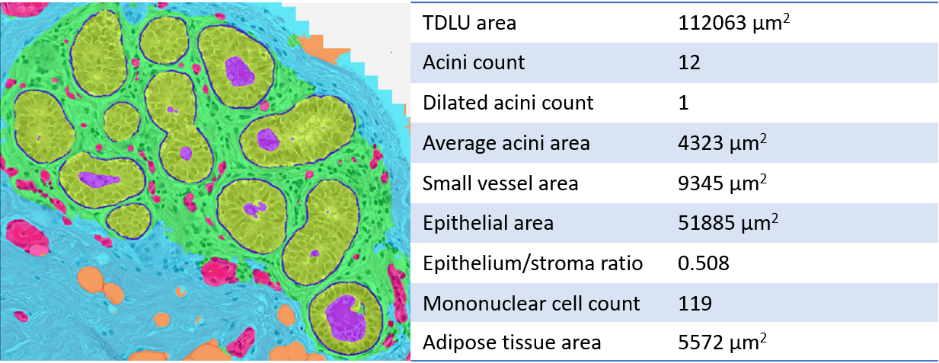


**Supplemental Figure 2: Forest plot showing comparisons of outcomes between parous and nulliparous women, where parous women were stratified according to time since last birth (≤5 years or >5 years). Effect sizes are in comparison to the nulliparous group and correspond to the multiplicative effect on the mean (TDLU number), the odds ratio for a higher acini number category (acini number), the multiplicative effect on the median (span), and the odds ratio for presence of the given outcome (BBD, plasma cells, inflammation). Effect sizes and 95% confidence intervals (CIs) result from multivariable regression models that were adjusted for age, race, BMI, percent fat, and relative with breast or ovarian cancer. ** Indicates a significant finding after applying a Bonferroni correction for multiple testing (P<0.0083). * Indicates a nominally significant finding (P<0.05) that did not survive Bonferroni correction for multiple testing.**

**
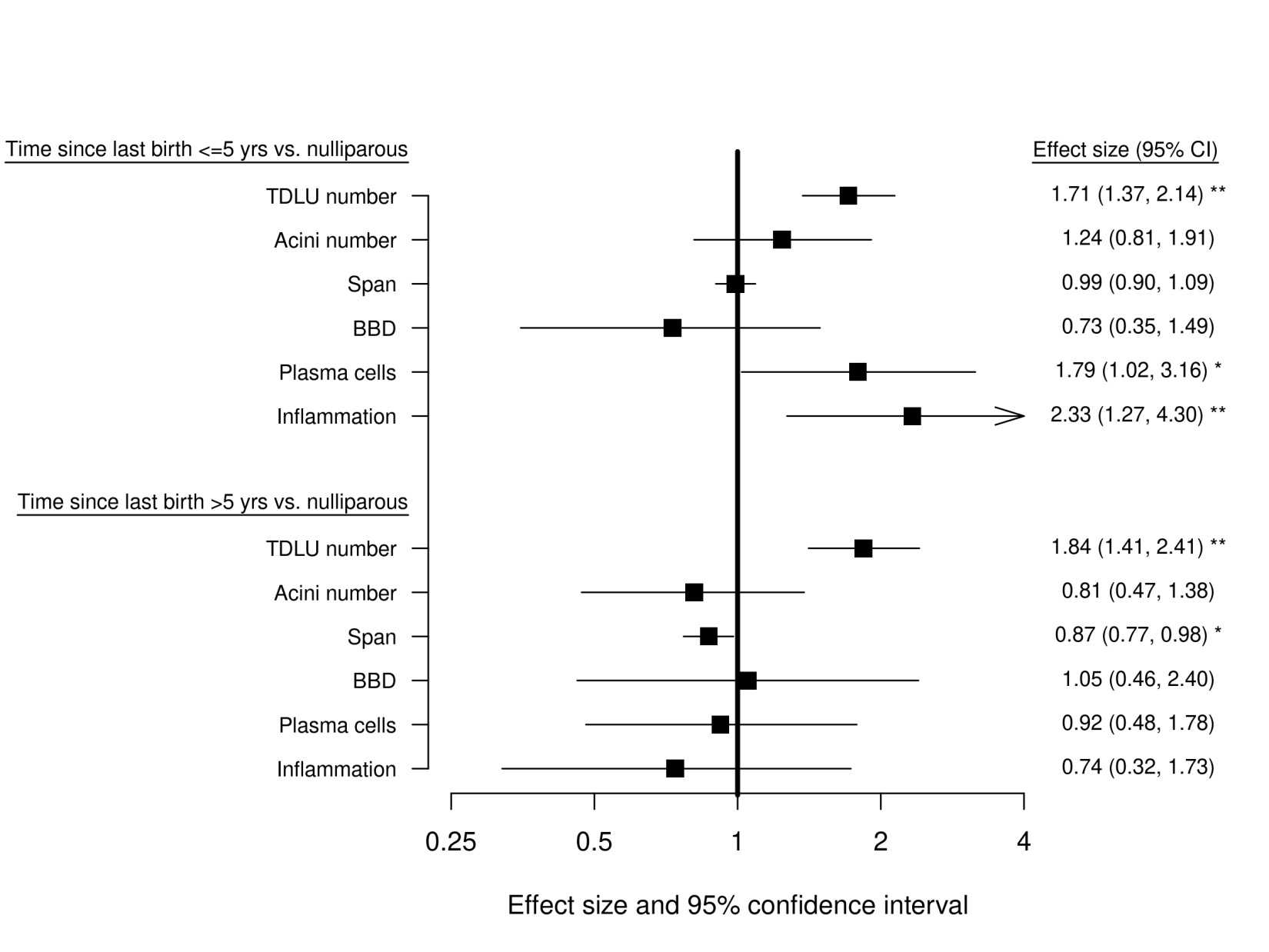
**
